# HIV and hepatitis B and C co-infection among people who inject drugs in Zanzibar

**DOI:** 10.1186/s12889-017-4933-0

**Published:** 2017-11-28

**Authors:** Ahmed Khatib, Eva Matiko, Farhat Khalid, Susie Welty, Ameir Ali, Asha Othman, Shaaban Haji, Mohammed Dahoma, George Rutherford

**Affiliations:** 1 Zanzibar AIDS Control Program, Ministry of Health, Zanzibar, United Republic of Tanzania; 2 Division of Global HIV/AIDS, US Centers for Disease Control and Prevention, Dar es Salaam, United Republic of Tanzania; 3 Global Health Sciences, University of California, San Francisco, San Francisco, CA USA; 4 Directorate of Preventive Services and Health Education, Ministry of Health, Zanzibar, United Republic of Tanzania

**Keywords:** Co-infection, Hepatitis B, Hepatitis C, Human immunodeficiency virus, Injecting drug users, Respondent-driven sampling, Zanzibar, Tanzania

## Abstract

**Background:**

People who inject drugs are at high risk of acquiring hepatitis B (HBV), hepatitis C (HCV), and human immunodeficiency virus (HIV) due to risky injection and sexual practices. The objective of this study is to investigate the epidemiology of HIV, hepatitis B, and hepatitis C, and co-infection of these viruses among people who inject drugs in Zanzibar, Tanzania.

**Methods:**

We used respondent-driven sampling to identify 408 participants, from whom we collected demographic data, information on sexual behaviours and injection drug practices, and blood samples for biological testing.

**Results:**

Prevalence of hepatitis B surface antigenaemia, HCV, and HIV infection were 5.9, 25.4, and 11.3%, respectively. Of the participants who were hepatitis B surface antigen (HBsAg) positive, 33.5% were infected with HCV and 18.8% were infected with HIV. Of the HCV-infected participants, 29.3% were infected with HIV. Of the participants who were infected with HIV, 9.0% were HBsAg positive, 66.6% had HCV and 8.5% had both. None of the potential risk factors we measured were associated with HBsAg positivity. In contrast, older age and longer duration of injection drug use were independently associated with HCV infection. HCV infection among people who inject drugs is lower in Zanzibar than in other countries, but could rise without proper interventions.

**Conclusions:**

These findings underscore the importance of screening people who inject drugs for HIV, HBsAg, and HCV; providing HBV vaccination to those who are eligible; initiating antiretroviral therapy for those who are co-infected with HIV/HBV and HIV/HCV; and introducing interventions that have high impact on reducing needle sharing.

## Background

An estimated 16 million people injected drugs worldwide in 2007 (range 11–21 million) [1]. People who inject drugs (PWID) have an elevated risk of acquiring parenterally transmitted infectious diseases, as the practice of sharing needles and other injection equipment can result in microtransfusions of blood and/or serum. There are an estimated 1.2 million PWID chronically infected with hepatitis B virus (HBV), 6.4 million with hepatitis C virus (HCV) [2–4], and 3 million with HIV [5]. Like HIV, HBV and HCV can be spread by unsafe injection practices [6], increasing the chances of PWID acquiring both viral hepatitis and HIV. It is estimated that between 5 and 25% of the HIV-infected persons also have HBV and/or HCV [7–9].

There are approximately 3000 PWID in Zanzibar [10]. Injection drug use is facilitated by its location on the Indian Ocean coast of Africa along drug trafficking routes [11]. Like in other East African coastal cities, investigators in Zanzibar have started reporting outbreaks of HIV infection associated with injection drug use [12].

In Zanzibar, the prevalence of HIV, HBV, and HCV among PWID has previously been reported as 16, 6.5, and 26.9%, respectively [13]. However, little is known about their burden of co-infection. Given that PWID are more vulnerable to blood-borne infections due to their risky injection practices, data on co-infection among this population is especially important. We used the opportunity of a recent HIV integrated bio-behavioural survey in Zanzibar to understand the epidemiology of HIV and HBV and HCV co-infection among PWID. To our knowledge, no studies have been conducted on this subject in Zanzibar.

## Methods

We conducted a cross-sectional survey to assess the prevalence of hepatitis B surface antigen (HBsAg) carriage and HCV seropositivity and associated risk behaviours among PWID on Zanzibar Island in 2012. Zanzibar Island is the main island of the Zanzibar archipelago, a semiautonomous region of the United Republic of Tanzania. Study methods have been described in detail elsewhere [10]; to summarize, we recruited male and female PWID aged 15 years and above, who had lived in Zanzibar the prior 3 months and who had injected illicit drugs in the prior 3 months. We used respondent-driven sampling (RDS), a social network-based recruitment methodology that allows weighted adjustment of key variables to produce unbiased representative results for difficult-to-reach and hidden populations [14, 15], to identify prospective study participants. We recruited six seeds, or initial participants identified through existing peer groups or organizations that work with PWID, to begin initial recruitment and added an additional three seeds in the fourth study week to increase recruitment rates; of the nine seeds, two were female and seven were male. We distributed 1235 coupons and recruited 518 potential participants. The longest chain in this survey was 21 waves. One hundred and nine individuals (21%) did not meet the eligibility criteria, and one (< 1%) did not consent to participate, resulting in a study population of 408 eligible PWID. All participants received $3.90 for completing the survey and providing a blood specimen and $1.30 for each successful recruit regardless of sex. Trained project staff assessed candidates’ eligibility for enrolment and collected demographic information and self-reported sexual and injection risk behaviour data through structured in-person interviews conducted by gender-matched interviewers. The network size was assessed by asking, “How many PWID do you know personally (i.e., who are living in Unguja, are aged 15 years and above, you know their name, you know who they are, and they know you)?” This was followed by a question asking how many of those they had seen in the past month, so the network size was based on the number they had seen in the past month.

Time from first injection was calculated by subtracting age at first injection from the participant’s current age, and this was assumed to be equivalent to their duration of injection drug use.

We based power and sample size estimates on achieving desired precision around point estimates for HIV infection in PWID. We based the HIV prevalence at 16%, with a 95% confidence interval of 11.6–21.6% leading to a sample size of 407, after correction for an expected design effect of 1.8, based on the literature available at the time of planning the study [16].

We tested blood serum for HIV antibodies using a serial algorithm in accordance with the national testing guidelines for HIV. We screened all specimens using Determine HIV1/2 test (Abbott Diagnostic Division, Hoofddorp, The Netherlands) and retested reactive specimens using Unigold (Trinity Biotech, Bray, Ireland). To ensure data quality, we conducted an external quality assessment and retested 10% of the non-reactive samples and all of the HIV reactive samples. We also retested the last test used in the field, followed by a series of ELISA tests, which were performed at the National Health Laboratory Quality Assurance and Training Center, the national reference laboratory in Dar es Salaam.

We tested blood serum for HBsAg using ACON HBsAg (ACON Laboratories, Inc., Hangzhou, China), a qualitative, lateral flow immunoassay for detection of HBsAg in serum or plasma. We measured antibody to HCV using ACON Hepatitis C (ACON Laboratories, Inc., Hangzhou, China) virus test strip, a qualitative, membrane-based immunoassay for the detection of antibody to HCV in serum or plasma. The Centers for Disease Control and Prevention’s Hepatitis Laboratory in Atlanta, USA, conducted external quality assurance (EQA) for HBsAg and HCV testing.

After all samples had been collected and tested locally, we stored all reactive and 10% of non-reactive samples at Mnazi Mmoja Laboratory at −20 °C prior to transportation to Atlanta. We conducted an external quality assessment once all samples had been collected and tested.

We used the Respondent-Driven Sampling Analysis Tool (RDSAT) 6.0.1, an open-source software package (www.respondentdrivensampling.org), to analyse data on the prevalence of HBsAg, HCV, and HIV; sexual and drug-related risk behaviours; demographic characteristics; and other variables, with adjustments for social network sizes and recruitment patterns. We calculated estimators and 95% confidence intervals for sexual and other risk factors associated with HBV and HCV transmission using partition and then prevalence analysis. We also used RDSAT to produce weights for the dependent variable used in multivariate analyses and exported these to STATA 12 (STATA Corporation, College Station, TX, USA) for bivariate and multivariate analysis using logistic regression. We selected variables that were associated with Hepatitis B or Hepatitis C infection in bivariate analysis at the level of *p* < 0.2. We then used backwards stepwise regression to find the best fit model as the one that included variables associated with HIV at or near the *p* < 0.05 level.

## Results

The sample was overwhelmingly male *N* = 401 (98%) and had a median age of 32 years. Overall, participants had a median duration of injection drug use of 5 years, 48% had injected for 3 years or less, and the median age at first injection was 26 years. Needle sharing was common; 54.8% had ever shared a needle, and 29% had shared a needle in the past month (Table 1).

**Table 1:** Sociodemographic characteristics, risk behaviours, and Hepatitis Co-Infection among PWID in Zanzibar, 2012

	Crude N	%^a^ [95% CI]
Age		
15–19 years	1	3.0 [0.0–10.0]
20–24 years	38	11.0 [7.1–15.7]
25–29 years	100	28.9 [22.7–35.5]
30–34 years	103	23.9 [18.8–30.4]
≥ 35 years	166	35.8 [28.9–41.7]
Median age in years (IQR) 32 years (IQR 28–38) Min. 18 – Max. 54		
Sex		
Male	401	98.5 [97.6–99.8]
Female	7	1.5 [2.0–2.4]
Income TZS among those who know their income		
< 50,000	1	0.6 [0.0–2.0]
50,000–120,000	17	12.4 [7.1–18.3]
120,001–200,000	18	10.6 [6.1–16.7]
≥ 200,001	187	76.5 [67.8–83.5]
Median income 450,000 TZS (IQR 300,000–600,000) Min. 3500 – Max. 12,000,000		
Duration of injection drug use		
3 years or less	167	48.0 [41.7–53.8]
4–6 years	76	15.1 [11.9–19.0]
7 years or more	165	36.9 [31.5–42.5]
Median duration	5 Years (IQR 2–9)	
Median age at first injection 26 years (IQR 21–30) Min. 12 – Max. 51		
Ever shared a needle	224	54.8 [48.5–61.0]
Injected in the last month with a needle previously used by someone else among all respondents	122	29.1 [23.6–36.2]
Injected blood from someone who had taken drugs in the past 1 month (flashblood)	19	4.8 [2.4–7.6]
Condom use at last sex with a non-paying partner among those who had a non-paying partner (*n* = 212)	68	14.4 [10.2–18.2]
Number of non-paid partners in past month among those who had a non-paid partner		
1 partner	117	86.4 [74.6–92.7]
2 or more partners	29	13.6 [7.3–25.4]
Median number of past month non-paid sex partners 1 partner (IQR 1–1) Min. 1 – Max. 5		
Frequency of condom use with non-paid partners in past month among those who had a non-paid partner		
Always	18	15.9 [8.3–23.0]
Inconsistently	34	13.1 [7.4–20.3]
Never	144	71.0 [62.4–80.0]
HBV	25	5.9 [3.5–8.8]
HBV and HCV (of those with HBV)	13	33.5 [16.2–56.9]
HBV and HIV (of those with HIV)	7	18.8 [7.3–40.4]
HCV	128	25.4 [19.1–32.0]
HCV and HIV (of those with HIV)	47	29.3 [21.1–39.1]
HIV	67	11.3 [7.7–15.2]
HIV and HBV (of those with HBV)	7	9.0 [2.3–19.3]
HIV and HCV (of those with HCV)	47	66.6 [52.3–83.0]
HBV and HCV and HIV (of those with HIV)	6	8.5 [1.8–18.6]

*CI* confidence interval, *IQR* interquartile range, *TZS* Tanzanian shillings

^a^RDSAT weighted

**Fig. 1:**
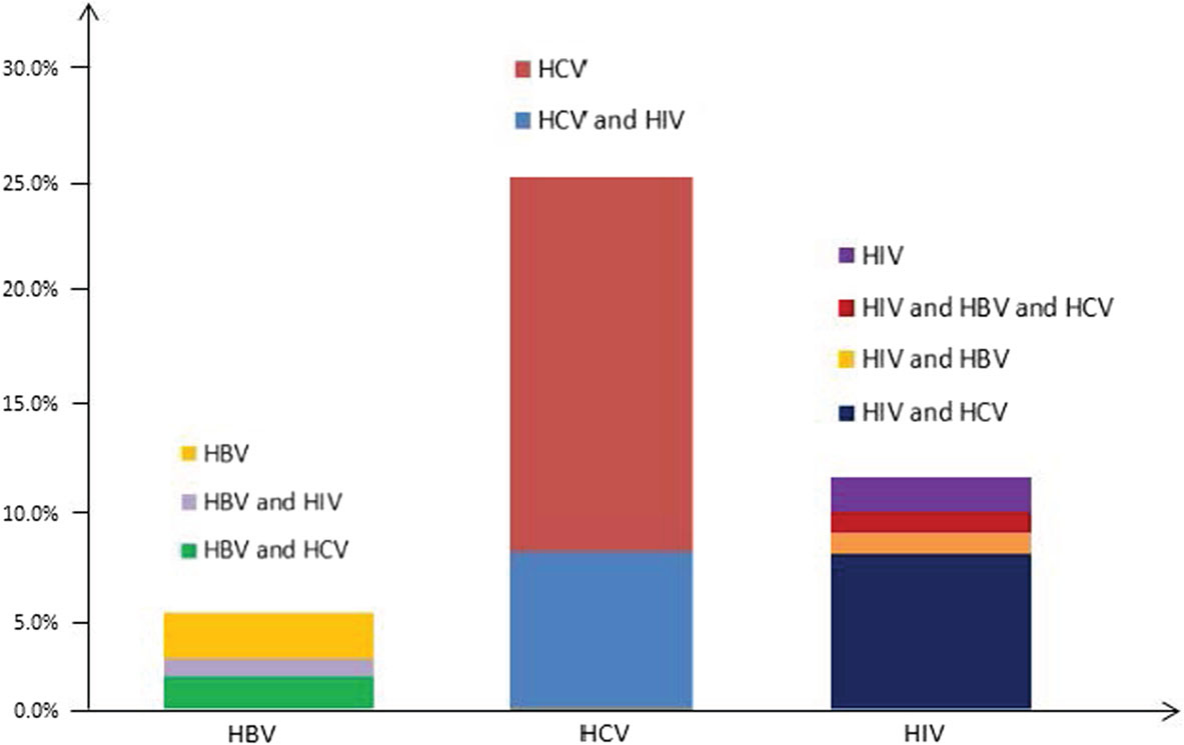
Rates of HIV, HCV, and HBV co-infection. Rates of HIV, HCV, and HBV co-infection among PWID in Zanzibar, Tanzania

An estimated 5.9% of participants were HBsAg positive, 25.4% were infected with HCV, and 11.3% were infected with HIV. Of the participants who were HBsAg positive, 33.5% were co-infected with HCV and 9.0% were co-infected with HIV. Of HCV-infected participants, 29.3% were co-infected with HIV. Of the participants who were infected with HIV, 9.0% were co-infected with HBV, 66.6% with HCV and 8.5% with both (Fig. 1). None of the potential injection risk factors measured were associated with HBsAg positivity (Table 2). In contrast, older age (above the median) and longer duration of injection drug use (above the median) were independently associated with HCV infection (Table 3).

**Table 2:** Predictors of Hepatitis B surface antigenaemia among people who inject drugs in Zanzibar, 2012

	Crude N	OR	*P* Value	95% CI
Age categories (above and below median age of 32)	204	1.33	0.24	0.83–2.12
Duration (above and below median of 5 year duration)	201	1.07	0.09	0.99–1.16
Income (Above and below 50,00 TShillings)	204	1.11	0.72	0.63–1.95
Ever shared a needle (No/Yes)	224	1.41	0.51	0.50–3.96
Shared a needle in last month (No/Yes)	112	1.47	0.53	0.44–4.92
Flash blood (No/Yes)	19	0.41	0.29	0.08–2.13
Had a paid partner in the last month (No/Yes)	93	0.46	0.15	0.16–1.33
Used a condom at last sex with paid partner (No/Yes)	97	1.03	0.95	0.47–2.24
Number of non-paying partners (above and below median number of 1)	117	0.31	0.30	0.03–3.74
Condom use with non-paid partner (No/Yes)	23	1.39	0.52	0.51–3.74

**Table 3:** Predictors of Hepatitis C among people who inject drugs in Zanzibar, 2012

	Crude N	OR	*P*-Value	95% CI	aOR	P- Value	95% CI
Age categories (above and below median age of 32)	204	2.52	0.00	1.44–4.39	2.06	0.02	1.14–3.73
Duration (above and below median of 5 year duration)	201	3.37	0.00	1.86–6.10	2.79	0.00	1.49–5.20
Income (Above and below 50,00 TShillings)	204	1.02	0.92	0.73–1.42			
Ever shared a needle (No/Yes)	224	1.66	0.07	0.96–2.89	1.63	0.09	0.93–2.84
Shared a needle in last month (No/Yes)	112	1.00	0.99	0.49–2.05			
Flash blood (No/Yes)	19	0.76	0.70	0.18–3.23			
Had a paid partner in the last month (No/Yes)	93	1.58	0.21	0.77–3.23			
Used a condom at last sex with paid partner (No/Yes)	97	1.21	0.36	0.81–1.80			
Number of non-paying partners (above and below median number of 1)	117	0.95	0.93	0.28–3.19			
Condom use with non-paid partner (No/Yes)	23	0.86	0.66	0.44–1.69			

## Discussion

We found that approximately one-fourth of PWID in Zanzibar were infected with HCV, which is moderately low compared to the same populations in other parts of the world [17–19]. HIV prevalence among PWID in Zanzibar was on the low end of the range for HIV prevalence in these same studies (4.6 to 59.6%). Chronic HBV infection (as measured by hepatitis B surface antigenaemia) was substantially less common among PWID in Zanzibar (5.9%). We also found that co-infection of HIV with HCV was substantial (66.6%). In addition, we found that infection with HCV was associated with age and duration of injection drug use, but were unable to associate standard risk factors for parenteral and sexual transmission with HBsAg positivity.

HCV among PWID has been widely described, but less so in Africa. In studies conducted in India, Iran, and Australia, investigators found HCV prevalence ranges from 34.5 to 90.4% [16–18]. Given their risky injection practices, it is surprising that PWID in Zanzibar have a relatively low prevalence of HCV compared to PWID elsewhere. It is likely that we have intervened in a moment where a larger HCV epidemic can be prevented. PWID may be a relatively new phenomenon in Zanzibar, or Zanzibari PWID may be more isolated from other countries’ networks. Prevention of HCV is difficult, and vaccine trials currently underway in other parts of the world may eventually benefit PWID in Zanzibar. The relatively low level of chronic HBV infection was expected and consistent with rates of perinatal transmission, understood through the recent introduction of prenatal screening for HBsAg and infant HBV immunization in Zanzibar. The background rate of HBsAg positivity in general African populations is estimated to be between 9 and 20% [20]; however, HBsAg prevalence in antenatal clinic surveillance (ANC) in Zanzibar was 1.3% [21]. Although this is low compared to the general population in other parts of Africa, given the relatively low HBV prevalence in the general population, the figure among PWID is high for Zanzibar though given that only surface antigen can be detected immunization coverage could account for this higher prevalence among PWID. Future studies would benefit from assessing the prevalence of hepatitis B core (HBcAb) and surface (HBsAb) antibody to understand the burden of disease and HBV immunization coverage.

Our study has some limitations. First, we measured HBsAg rather than HBV antibodies; therefore it is impossible to distinguish chronic HBV infection from acute HBV infection. In addition, self-reported behavior, recall bias, and social-desirability bias may have influenced participant’s responses. Underreporting of these risks may have biased results toward the null with consequent Type II error. Secondly, we used RDS to generate our study population, which is highly dependent on accurate estimates of participants’ network sizes [14]. Inaccuracies in estimating network sizes can lead to overestimation or underestimation of outcome variables. However, a particular strength of RDS is its ability to enroll a diverse range of participants from different social networks [13, 14]. How well these social networks intersect by the end of the survey is among the more important determinants of the accuracy of the estimates produced.

Interestingly, despite having two female seeds, very few female PWID were enrolled in the survey. Other similar studies have had difficulty enrolling female PWID using RDS [22], though studies have been successful at recruiting female PWID using convenience-based sampling techniques at HIV counseling and testing centers [23]. It may be the case the female PWID are not as accessible through peer referral as male PWID and they may need to be sought out in order to participate in surveys. It is also possible that there are fewer female PWID.

## Conclusions

Despite these potential limitations, we conclude that HCV infection is common among PWID in Zanzibar, although at a much lower prevalence than in PWID in more mature epidemics. With improved access to prevention, HCV prevalence may stabilize and eventually decline. HBV prevalence among PWID is also low compared to PWID in other settings. This underscores the importance of screening PWID for HIV, HBV, and HCV; providing HBV vaccination to those eligible; initiating antiretroviral therapy early for those with HIV/HBV or HIV/HCV co-infection; introducing interventions that have high impact on reducing needle sharing; and continued monitoring to understand the impact of prevention programs.

## Abbreviations

CI: Confidence interval
HBsAg: Hepatitis B surface antigen
HBV: Hepatitis B virus
HCV: Hepatitis C virus
HIV: Human immunodeficiency virus
IQR: Interquartile range
PWID: People who inject drugs
RDS: Respondent-driven sampling
TZS: Tanzanian shillings
